# A New Nasal Restriction Device Called FeelBreathe^®^ Improves Breathing Patterns in Chronic Obstructive Pulmonary Disease Patients during Exercise

**DOI:** 10.3390/ijerph17134876

**Published:** 2020-07-06

**Authors:** José L. Gonzalez-Montesinos, Aurelio Arnedillo, Jorge R. Fernandez-Santos, Carmen Vaz-Pardal, Pelayo A. García, José Castro-Piñero, Jesús G. Ponce-González

**Affiliations:** 1 Department of Physical Education, Faculty of Education Sciences, University of Cádiz, 11519 Cádiz, Spain; jgmontesinos@uca.es; 2 University Hospital Puerta del Mar. Pneumology, Allergy and Thoracic Surgery Department, 11009 Cádiz, Spain; aurelioarnedillo@neumosur.net; 3 Biomedical Research and Innovation Institute of Cádiz (INiBICA) Research Unit, 11009 Cádiz, Spain; jose.castro@uca.es (J.C.-P.); jesusgustavo.ponce@uca.es (J.G.P.-G.); 4 GALENO Research Group and Department of Physical Education, Faculty of Education Sciences, University of Cádiz, 11519 Cádiz, Spain; 5 Bahía Sur Andalusian Center for Sports Medicine, 11100 Cádiz, Spain; carmenvaz@hotmail.com; 6 Center Sport Iberia, 29007 Málaga, Spain; pelayo.arroyo@uca.es; 7 MOVE-IT Research Group and Department of Physical Education, Faculty of Education Sciences, University of Cádiz, 11519 Cádiz, Spain

**Keywords:** COPD, respiratory muscle training, exercise

## Abstract

A device called FeelBreathe (FB)^®^ was designed, developed, and patented for inspiratory muscle training. The main aim was to determine the acute responses on lung ventilation, gas exchange, and heart rate during exercise in patients with chronic obstructive pulmonary disease (COPD) with and without the use of FB. In this study, a randomized cross-over trial was performed with 18 men diagnosed with COPD (FEV_1_ between 30% and 70% of its predicted value). Each participant randomly conducted two trials with 30 min of rest between them with the same protocol on a treadmill for 10 min at a constant rate of 50% of VO_2peak_. Each test was performed randomly and in a crossover randomized design in two different conditions: (1) oronasal breathing; and (2) nasal breathing with FB (nasal ventilatory flow restriction device). It was observed that FB had positive effects on dynamic hyperinflation, breathing pattern, and breathing efficiency, with higher expiratory and inspiratory time. Despite these differences, blood oxygen saturation percentage, oxygen uptake, and heart rate showed a similar response for both conditions during exercise. The results suggest that exercise performed with FB improved ventilatory responses compared to the oronasal mode in COPD patients. This new tool could be used during most daily tasks and exercise programs.

## 1. Introduction

Chronic obstructive pulmonary disease (COPD) is a pulmonary disorder characterized by the presence of inflammation, producing obstruction of the airways, deterioration of the quality of life, dyspnea, and intolerance to physical exercise [1], due in part to impairment of muscular strength and resistance and to dynamic hyperinflation, associated with a possible premature death [2]. Respiratory muscle training is considered of vital importance, since it could improve the exercise capacity, dyspnea, and quality of life of patients with cardio-pulmonary disease [3,4]. Specifically, inspiratory muscle training (IMT) has been proved to induce an effective improvement of maximal inspiratory pressure (P_Imax_) and perception of well-being in COPD patients [3], and health-related quality of life in patients with chronic heart disease, respiratory disease, and breathlessness during exercise [3,5]. The most popular IMT methods used in COPD are resistive loading, pressure threshold loading and voluntary normocapnic hyperpnea [5,6], although the devices involved must be used in static positions, with a nose clip and breathing though the mouth (atypical breath) [3]. New comfortable devices for respiratory muscle training that could be used in dynamic conditions, such as walking or performing daily life activities, should be investigated in COPD patients.

A nasal ventilatory flow restriction device called FeelBreathe (FB) was designed, developed, and patented to increase nasal airflow resistance (Supplemental file S1). Previous studies with healthy subjects have shown that FB causes improvements in lung ventilation, ventilatory efficiency, and heart rate during exercise [7], which could be a target of respiratory muscle training in COPD. An increased airflow resistance while breathing nasally during exercise increases the breathing effort [8], which may potentially improve exercise tolerance and energy efficiency [9]. The main aim of the present study was to examine the effects of FB in lung ventilation, gas exchange and heart rate during exercise in patients with chronic obstructive pulmonary disease.

## 2. Materials and Methods

### 2.1. Sample Size

The sample size needed was calculated using the G*Power version 3.1 software [10]. According to the results of the F tests we needed a sample of 20 participants in order to obtain a significant difference between oronasal breathing (ONB) and FB conditions (Statistical test: ANOVA-Repeated measures, within-between interaction. Required input parameters: effect size = 0.25, level of significance α = 0.05, power β = 0.80, number of groups = 2; Number of measurements = 10; Correlation between repeated measures = 0.5, nonsphericity correction ε = 1). Effect size was obtained from the study performed by González-Montesinos et al. [7].

### 2.2. Participants

**Figure 1:**
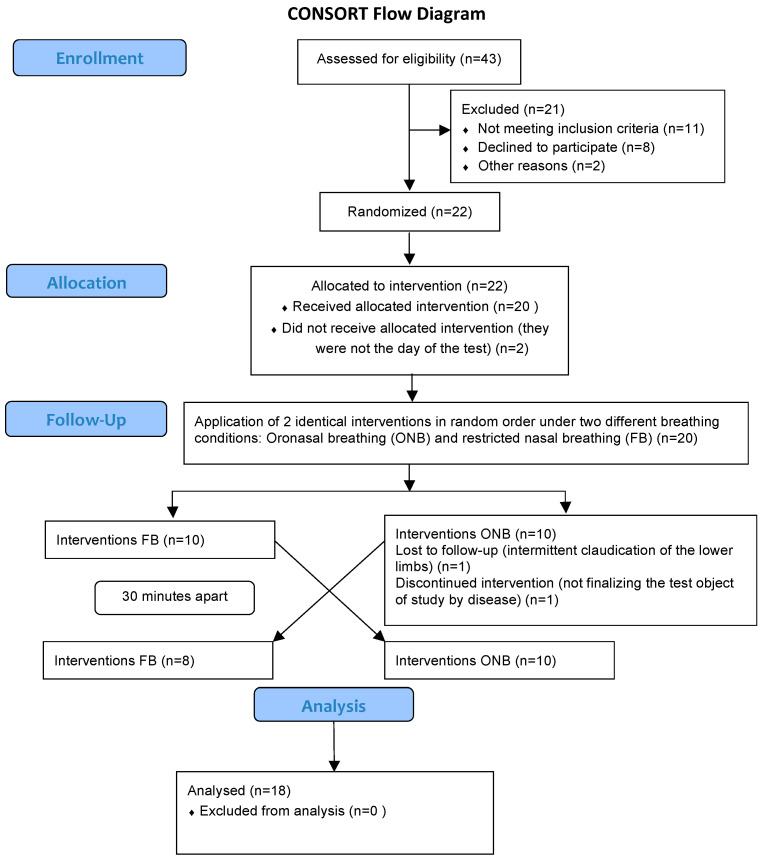
Illustration of the design and analysis of the cross-over trial.

Forty-three participants were recruited from the University Hospital Puerta del Mar. The inclusion criteria were: men aged between 40 and 70 years, diagnosed with COPD at least 6 months before, with a stable clinical situation and with a forced expiratory volume in the first second (FEV_1_) between 30% and 70% of the predicted value, according to the criteria of the ATS [11]. This interval was chosen because dyspnea is rarely present above 70%, and patients with FEV_1_ below 30% cannot perform the tests due to their clinical situation. Key exclusion criteria included COPD exacerbations that required treatment with antibiotics or oral steroids or hospitalization in the 8 weeks prior to inclusion, other medical conditions that contribute to dyspnea on exertion, or diseases that may interfere with the realization of the exercise test. Twenty-two patients were included in the study, although only twenty of them were randomized since two patients could not attend the day of the tests. Moreover, two patients were excluded: one of them due to intermittent claudication of the lower limbs and the other one for not finalizing the test due to disease. Therefore, eighteen patients completed all of the tests (Figure 1). A written informed consent was obtained from all the patients before starting the study. This clinical trial received ethical approval from the Ethics Committee of the University Hospital Puerta del Mar and met the requirements of the Declaration of Helsinki.

### 2.3. Study Design

A cross-over randomized design was used. Randomization of the participants to ONB or FB condition in a 1:1 ratio was performed by the package randomizeR for R using a complete randomization process [12].

On the first day, after explaining the study, the patients signed the informed consent and performed the pulmonary function tests in the University Hospital Puerta del Mar. The rest of the tests were completed and conducted on different days. All testing sessions were performed under similar environmental conditions (20–24 °C, 45–55% relative humidity).

On the second day, the patients completed a health questionnaire, performed a resting electrocardiogram (QRS Universal ECG, Plymouth, Massachusetts USA) and a spirometry (spirometer CPX, Cardinal Health, Hoechberg, Germany) according to criteria published by the ATS (Celli et al. 2004). In addition, PImax was measured during a maximal, static inspiratory effort (Micro Medical Ltd., Chatham, Kent, UK). PImax was recorded as the highest value averaged over 1 s from three maneuvers that varied by less than 10% and was measured based on three maximal reproducible respiratory efforts [11]. Finally, an incremental test on treadmill (Technogym Run Race 1400HC, Gambettola, Italy) was performed to determine the $\dot{V}$O_2peak_ using Balke protocol (Cardinal Health 234 GmbH, Leibnizstrasse 7, D-97204 Hoechberg, Germany). While performing the tests, an electrocardiogram and heart rate was measured every 10 s (JECG 12 Canal, Friedberg, Germany). Additionally, blood pressure was measured at the end of the test. Blood oxygen saturation percentage (Ear oximeter, Hewlett-Packard 47201A, Corvallis, OR, USA) and respiratory gas exchange were measured every 5 s and breath by breath, respectively, throughout the test.

On the third day, each patient randomly conducted two identical walking exercise tests for 10 min at a constant rate of 50% $\dot{V}$O_2peak_ on a treadmill after 1 min of warming up at 3 km/h, with 30 min of rest between sets in a sitting position [13]. Each test was performed under two different breathing conditions: (1) oronasal breathing (ONB trial); and (2) restricted nasal breathing with FB (FB trial). None of participants showed any respiratory difficulties at rest with FB. They felt an increase in respiratory effort due to a decrease in airflow only during exercise with FB.

During each walking exercise test, the patients were connected to the gas analyzer with a Hans Rudolph nasal/oral, two-way and non-rebreathing face mask (model 8900, Kansas City, MO, USA), which covered the subject’s mouth and nose. The inner seal of the mask was removed to ensure that the mask did not impinge on the nares during the tests. For ONB, the patients were asked to breath normally, freely. The following variables respiratory were collected during the walking exercise test: inspiration time (Tin); inspiratory time fraction (Tin/Tot); expiratory tidal volume (VTex); fraction of expired carbon dioxide (F_E_CO_2_); oxygen uptake (VO_2_); expiration time (Tex); inspiratory tidal volume (VTin); carbon dioxide production (VCO_2_); heart rate (HR); end-tidal partial pressure of carbon dioxide (P_ET_CO_2_); fraction of expired oxygen (F_E_O_2_); expiratory time fraction (Tex/Tot); breathing frequency (B_F_); deadspace/tidal volume ratio (V_D_/V_T_); respiratory quotient (RER); ventilatory equivalent ratio for carbon dioxide (EqCO_2_); ventilatory equivalent for oxygen (EqO_2_); end-tidal partial pressure of oxygen (PETO_2_); and minute ventilation (V_E_).

### 2.4. Statistical Analysis

Descriptive results are presented as mean ± standard deviation. Before any analysis, the cardioventilatory variables were classified according to our expected hypothesis as those with higher values in the FB trial (FB > ONB; i.e., Tin, Tin/Tot, Tex, VTex, VTin, FECO_2_, PETCO_2_, VO_2_, HR and VCO_2_) and those with higher values in the ONB trial (ONB > FB; i.e., RER, B_F_, EqO_2_, FEO_2_, Tex/Tot, PETO_2_, V_E_, EqCO_2_ and VD/VT). The proportion of responders and non-responders was calculated for each of the cardioventilatory variables using the typical error (TE) [14], and it was obtained from the percentage of change of the participants (%TE). Responsiveness was defined as a change beyond ± 1%TE. Additionally, a one-sample proportion test was performed to assess that the probability of to be a responder was greater than 50%. Differences between FB and ONB conditions in P_Imax_ and rating of perceived exertion (RPE) post-tests were analysed by *t*-test for paired samples. The values of the cardioventilatory variables obtained from the maximal incremental exercise tests were analysed using Bayesian functional ANOVA [15]. Since we are specifically interested in the effect of FB, a one-way functional ANOVA was fitted:

$$y_{i,j}\;|\;\mu ,\;\alpha_{i},\;\sigma^{2},∼N\left(\mu \;+\;\alpha_{i},\;\tau \right)$$

$$\mu ∼\mathrm{RW}2\left(\theta_{\mu }\right)$$

$$\alpha_{i}∼\mathrm{RW}2\left(\theta_{\alpha }\right)$$

where $y_{i,j}$ denotes the realization of the cardioventilatory variable for ith breathing condition (i.e., ONB or FB) from the jth participant, μ is the grand mean function, and $\alpha_{i}$ is the main effect function for the ith breathing condition. $\alpha_{\mathrm{ONB}}$ = 0 for identifiability. μ and $\alpha_{i}$ follow a previous second-order random walk prior for equally-spaced locations (RW2). Finally, a scaled hyperprior was set on τ via the scale model argument in the INLA formula. This model was fitted using the package brinla for R, which internally makes use of INLA [16]. Once the model was fitted, we analyzed the set of temporal points (i.e., excursion set) of the walking exercise test where there was a significant effect of FB via the package excursions [17], which is called internally by brinla. We used a numerical integration method to find the excursion set due to the Gaussian likelihood and the small sample size. VO_2_ and VCO_2_ were log-transformed to compute the Bayesian functional ANOVA, thus the results of these variables are shown in log-scale. All the analyses were performed with the R software environment for statistical computing [18] and can be found in https://github.com/JorgeDelro/COPD_1.3.

## 3. Results

The descriptive characteristics of the sample, pulmonary function, P_Imax_ and Borg’s scale recorded after the submaximal tests are displayed in Table 1. The rating of perceived exertion after the maximal incremental exercise test was significantly higher in the FB trial (*p* < 0.01, Cohen´s d = 0.61).

**Figure 2:**
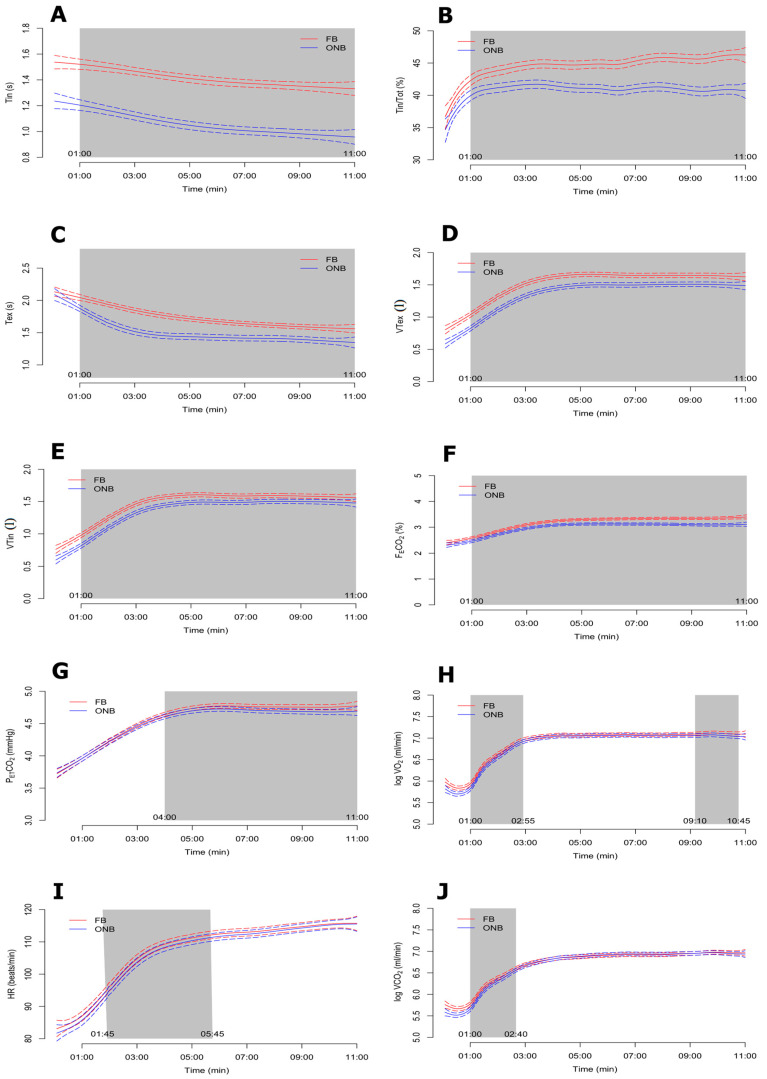
Bayesian functional ANOVA plots for FB > ONB group variables: inspiration time (Tin) (**A**), inspiratory time fraction (Tin/Tot ) (**B**), expiration time (Tex) (**C**), expiratory tidal volume (VTex); (**D**), inspiratory tidal volume (VTin) (**E**), fraction of expired carbon dioxide (FECO_2_) (**F**), end-tidal partial pressure of carbon dioxide (PETCO_2_) (**G**), oxygen uptake (VO_2_) (**H**), heart rate (HR) (**I**) and carbon dioxide production (VCO_2_) (**J**).

**Figure 3:**
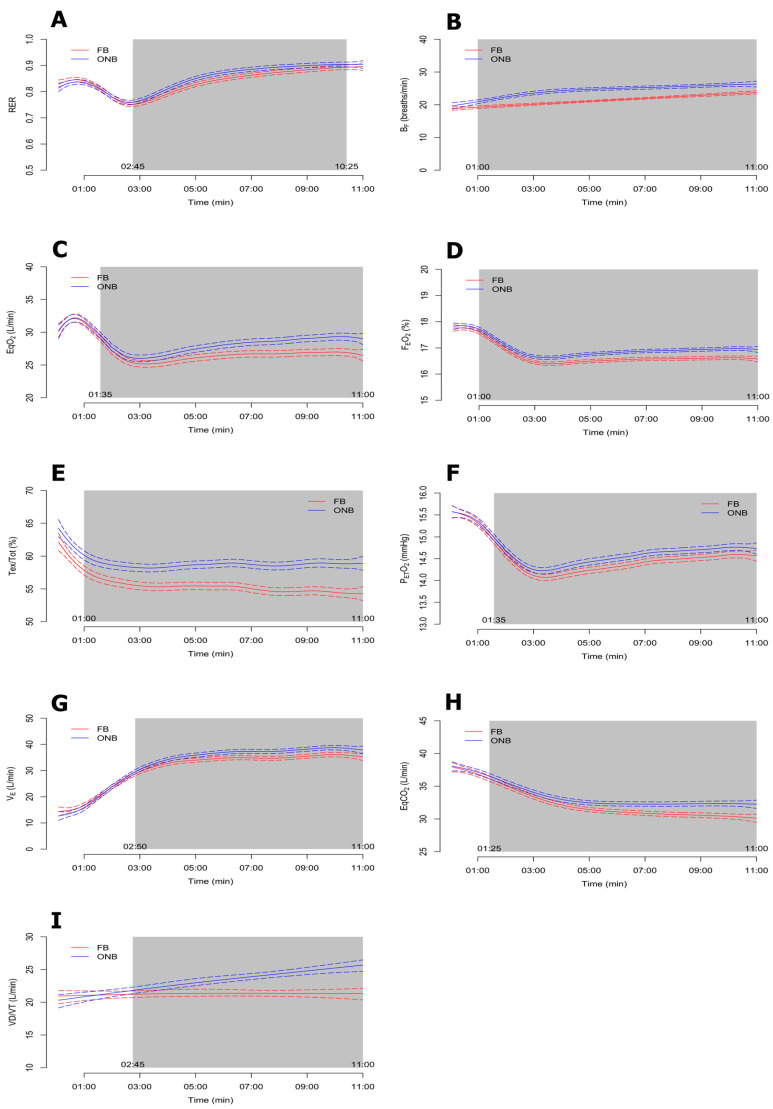
Bayesian functional ANOVA plots for ONB > FB group variables: respiratory quotient (RER) (**A**), breathing frequency (B_F_) (**B**), ventilatory equivalent ratio for carbon dioxide (EqCO_2_) (**C**), fraction of expired oxygen (FEO_2_) (**D**), expiratory time fraction (Tex/Tot), (**E**), end-tidal partial pressure of oxygen (PETO_2_) (**F**), minute ventilation (V_E_) (**G**), ventilatory equivalent ratio for carbon dioxide (EqCO_2_) (**H**) and dead space/tidal volume ratio (VD/VT) (**I).**

The Bayesian functional ANOVA results for each of the cardioventilatory variables are displayed in Figure 2 and Figure 3, respectively. All the analyzed variables showed significant differences between ONB and FB trials at some point of the test, except VO_2_ and VCO_2_, which had differences only at the beginning of the test. The results showed that breathing patterns were more efficient when the tests were performed with FB compared to ONB.

**Figure 4:**
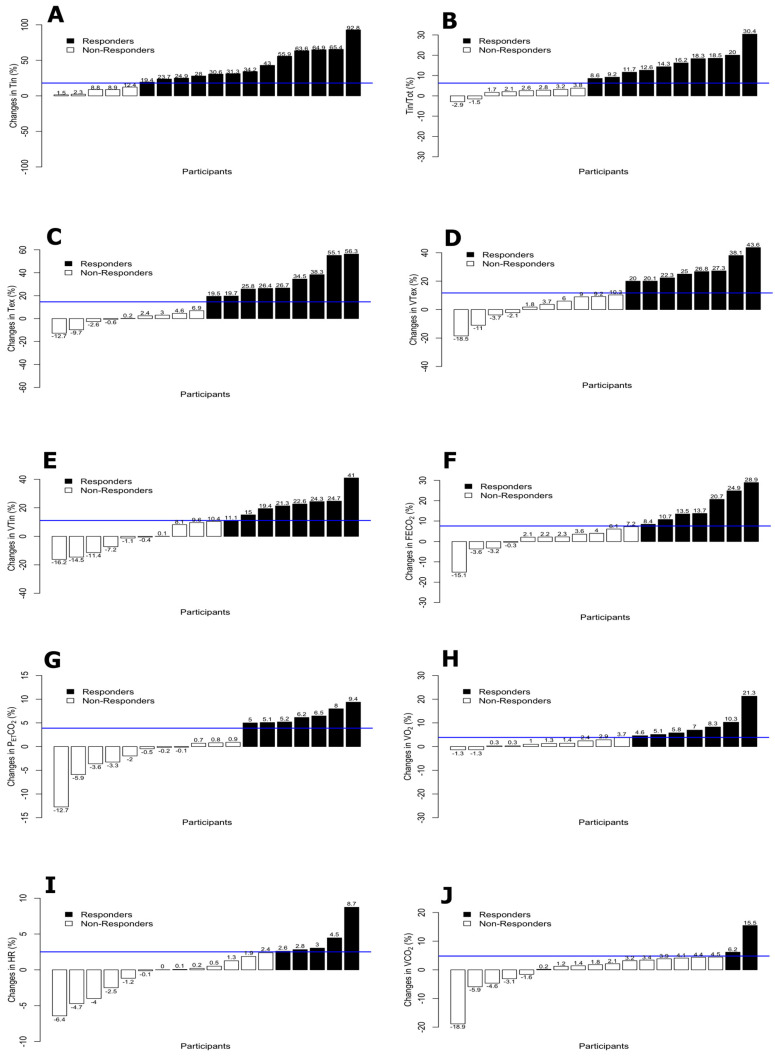
Percentage of change by participants and TE for FB > ONB group variables: Tin (**A**), Tin/Tot (**B**), Tex (**C**), VTex (**D**), VTin (**E**), FECO_2_ (**F**), PETCO_2_ (**G**), VO_2_ (**H**), HR (**I**) and VCO_2_ (**J**). Responders vs. non-responders on cardioventilatory variables with higher values in FB.

**Figure 5:**
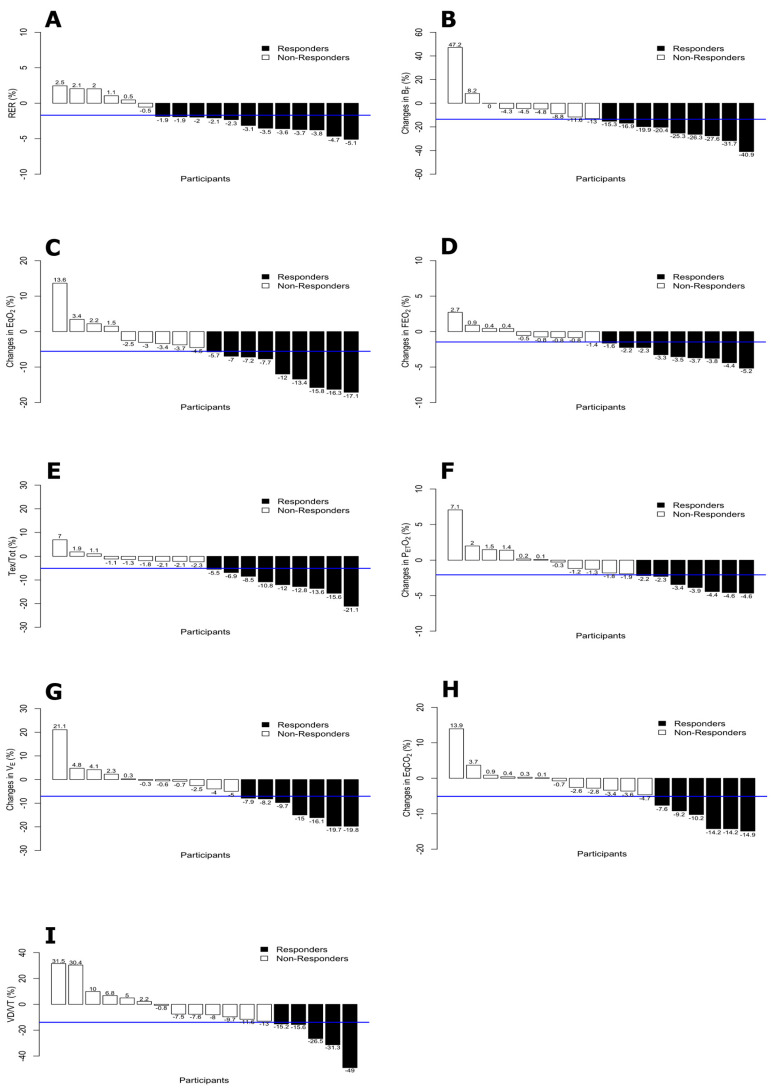
Percentage of change by participants and TE for ONB > FB group variables: RER (**A**), B_F_ (**B**), EqO_2_ (**C**), FEO_2_ (**D**), Tex/Tot (*E*), PETO_2_(**F**), VE(**G**), EqCO_2_ (**H**) and VD/VT (**I**).

The means, standard deviations and proportions of responders and non-responders for the cardioventilatory variables are shown in Table 2. Tin and VCO_2_ were the variables with higher and lower proportion of responders respectively (72.2% and 11.1%). Additionally, the percentage of change by participants for each of the variables are displayed in Figure 4 and Figure 5.

Considering the use of FB during the tests and all the outcomes related to dynamic hyperinflation (Tin, TinTot, Tex, and VTex and VTin), the number of responders was over 50% of the participants, but only in Tin was the proportion statistically significant (*p* < 0.05). Moreover, the variables related to breathing pattern (VE, VT, and BF) obtained responders in more than 44% of the participants, while breathing efficiency (Tin, TinTot, EqO_2_, and EqCO_2_) obtained responders in more than 52% of the participants. Finally, the expiratory and inspiratory time (Tin, TinTot, Tex and TexTot) and the parameters of effort intensity (HR and RER) obtained responders in more than 56% and 47% of the participants, respectively.

The largest numbers of responders occurred in Tin (13/18 participants, 5 participants with a percentage of change higher than 50%) and RER (12/18 participants). Moreover, the variables Tin/Tot, Tex, BF, EqO_2_, FEO_2_, and Tex/Tot showed a number of responders greater than or equal to 50% of the participants.

## 4. Discussion

The main finding of the study was that the FB device is a possible effective tool for inspiratory muscle training, since this study shows that FB caused higher expiratory and inspiratory time and increased the efficiency of breathing patterns compared to ONB in a group of COPD patients.

### 4.1. Effect of FB on Dynamic Hyperinflation

Dynamic hyperinflation is the main clinical cause of exertional breathlessness in patients with COPD [19]. Puente-Maestu et al. [20] observed that physical exercise has beneficial effects on respiratory patterns and dynamic hyperinflation, which may partially explain the improvements on dyspnea and exercise intolerance. Moreover, we showed that using FB during exercise reduced B_F_ and increased the inspiratory fraction, which may increase the effects of exercise on dynamic hyperinflation and the sense of breathlessness mentioned above [3,21,22]. Using FB, the values of B_F_ and minute ventilation $\dot{V}$_E_ (Table 2) decreased from minute 1 to 11 (Figure 3B) and from minute 2:50 to 11, respectively (Figure 3G), while inspiratory time, expiratory time and tidal volume increased, from minute 1 to 11 in all cases (Figure 2C). However, Tex/Tot was lower in FB from min 1 to 11 (Figure 3E). Moreover, the number of responders was greater than 50% of the participants with FB.

These results may suggest a positive effect of FB on dynamic hyperinflation in patients with COPD during exercise. Moreover, FB could optimize breathing patterns during exercise, which hypothetically could reduce dynamic hyperinflation [3].

### 4.2. Effect of FB on Breathing Patterns

Petrovic et al. [3] observed that one of the effects of IMT was a significant decrease in the ratio of breathing frequency to minute ventilation (B_F_/$\dot{V}$_E_), indicating an improved breathing pattern. In line with the results of Petrovic et al. [3], our study showed lower B_F_/$\dot{V}$_E_ in FB compared to ONB during exercise (0.66 versus 0.72). This could be explained by the fact that the patients adopted a slower, deeper breathing pattern during exercise due to the airflow restriction with FB. Moreover, FB causes nasal inspirations and mouth exhalations, improving the humidification, heating, and filtering of the air, as it represents a normal mechanism of heat and moisture exchange in the respiratory tract [23].

### 4.3. Effect of FB on Breathing Efficiency

There was a lower $\dot{V}$_E_ with FB and a light increase in $\dot{V}$O_2_ (Table 2) during the first two minutes and during the last minute of the exercise test (Figure 2H), without any changes in SpO2 (~96.8%, data not shown) compared to ONB. Moreover, two parameters related to breathing efficiency and O_2_ dynamics (EqO_2_ and EqCO_2_) were lower with FB (Table 2; Figure 3C,H). This result might be due to the improvement of gas exchange, producing a lower dynamic hyperinflation, as mentioned above. Similarly, the FB trial showed lower mean values of FEO2 (Figure 3D), with positive effects in 14/20 participants, while FECO_2_ increased throughout the test, which corresponds to a concomitant rise of oxygen utilization by the cells. Moreover, we observed a large functional reserve in the muscles at the end of an incremental exercise, regardless of the inspiratory O_2_ pressure, since it seems that $\dot{V}$O_2_ uptake is primarily dependent on convective O_2_ delivery and less limited by diffusion constraints [24,25]. Therefore, FB requires deeper, slower, and more O_2_-extracting breaths, inducing more effective breathing patterns.

It was shown that nasal breathing alters the dynamics of air flow in the upper respiratory tract and influences gas absorption compared to oronasal breathing [26]. Using FB during exercise produces a change in the breathing pattern while also producing an increase in the respiratory effort with respect to oronasal breathing.

There are systems which cause external thoracic restriction that have been used in healthy participants to simulate restrictive ventilator disorders, which lead to a rapid, shallow pattern of breathing sometimes associated with alveolar hyperventilation [27,28], high inspired minute ventilation, and low PETCO_2_, suggesting a higher level of alveolar ventilation [29]. The activation of inspiratory muscles with FB could reduce these symptoms, since FB seems to improve the breathing pattern, with a reduction of minute ventilation and an increment of PETCO_2_ favoring more efficient breathing.

### 4.4. Effect of FB on Expiratory and Inspiratory Time

The higher VT values found in FB vs. ONB could be explained by the higher % of time for inspiration and expiration with FB. Higher Tin, VTin, Tex, and VTex (Figure 2A–E) involved a greater respiratory effort using FB. It was shown that changes in the breathing timing could improve the dynamic pathophysiology in COPD [30]. The time of activation of the diaphragm and other respiratory muscles is greater with respiratory muscle training devices, which produce a greater voluntary hyperpnea [31]. However, this did not occur with FB, since the amount of air going into the lungs was higher compared to ONB, although the participants spent more time for inspiration (Tin). This effect occurred in most of the participants (13/18), who were considered as responders with FB for Tin. The last effect combined with a lower respiratory B_F_ rate caused a lower $\dot{V}$_E_ with FB (Table 2). Tin/Tot was greater with FB throughout the test (Figure 2B), which implies an increase in the inspiratory musculature activation time, producing a greater performance of this musculature using FB.

Due to the increase of Tin with FB, more time would be used to perform the expiratory phase, as reflected by the 11.7% increase in Tex. However, Tex/Tot was lower with FB (−6.2%), which means an increase in expiratory effort, despite the fact that the amount of air was higher with FB (+12.5% for VTex), and this situation occurred during the entire test (Figure 2D and Figure 3E). Similarly, it was shown that expiratory muscle training for deep breathing with adequately prolonged inspiration during exercise may increase exercise tolerance [30,32]. Therefore, this suggests that FB could be used as a new system of RMT, for both inspiratory and expiratory muscles.

### 4.5. Effect of FB on Intensity

We observed a significant difference in heart rate between FB and ONB test during the first five minutes (Figure 2I), but not in the final minutes of exercise testing. This response might be normal, since the patients had to get used to the FB at the beginning of the exercise; however, the HR average of the entire test was similar for both conditions. COPD patients may have associated cardiac pathologies which do not recommend exercises that raise HR in excess [33].

Differences in RER were observed between FB and ONB (Figure 3A), with lower values in FB after the first minutes of stabilization (Table 2), despite the absence of differences between conditions in VO_2_ and VCO_2_ kinetics during the test. A possible explanation is that V_E_ in the FB trial was reduced compared to ONB, which could also reduce RER by slight changes on VO_2_ and VCO_2_.

Inspiratory muscle fatigue caused by airflow restriction during exercise increases sympathetic vasomotor outflow, which produces an increase in PETO_2_, as variable of fatigue [34]. However, our results showed slightly lower values of PETO_2_ in FB trial with differences from min 1:35 (Figure 3F). Lower PETO_2_ could reduce SpO_2_ and the dissociation of O_2_ from hemoglobin into the cells.

The rating of perceived exertion RPE Post-FB showed a slightly greater sense of force, without becoming, at any time, a strenuous force of the inspiratory musculature and with similar heart rate values throughout the whole FB vs. ONB test.

## 5. Limitations of the Study and Perspectives of Future

The main limitation of this study was the relatively small sample size (18 participants included in the analysis), so results must be taken with caution. However, there are several recent articles on exercise effects in COPD patients with a similar sample sizes [35,36,37,38,39]. To minimize the sample size problem, Bayesian statistics was used in this study which may produce reasonable results even with small samples. Moreover, the nasal breathing condition was not included, due to the fact that a previous study with healthy cyclists showed that nasal breathing was between FB and ONB in the most of the variables of gas exchange [7]. In future, exercise interventions combined with FB should be investigated to clarify the chronic effects on breathing patterns in COPD patients.

## 6. Conclusions

FB is a new device for training ventilatory muscles during exercise in COPD patients, since it produces lower dynamic hyperinflation and improved breathing patterns and efficiency, with higher expiratory and inspiratory time compared to natural breathing. Our findings provide a new tool that could be used during most daily tasks and exercise with a natural breath and simple shape, unlike other IMT devices.

## Figures and Tables

**Table 1:** Descriptive characteristics and pulmonary function of the sample. Values of P_Imax_ and RPE after the 10 min walk test without FeelBreathe (post oronasal breathing (ONB)) and with FeelBreathe (post FB).

Variables	Pre	Post-ONB	Post-FB
Age (years)	67.5 (3.0)		
Weight (kg)	77.1 (11.1)		
Height (cm)	167.4 (7.5)		
BMI (kg/m^2^)	27.1 (3.6)		
VO_2_peak (mL/kg/min)	20.8 (5.3)		
FVC (mL)	2874.6 (772.5)		
FVC (%)	61.8 (22.2)		
FEV_1_ (mL)	1770.7 (540.7)		
FEV_1_ (%)	57.1 (14.2)		
FEV_1_/FVC	61.6 (7.9)		
PImax (cmH_2_O)	102.3 (25.2)	100.5 (27.6)	95.2 (27.3)
RPE scale (Borg units)		10.2 (2.9) **	11.9 (2.6)

Descriptive values are shown as mean (standard deviation). ** *p* < 0.01 for differences between RPE Post-ONB and RPE Post-FB (Cohen’s d = 0.61). FVC: forced vital capacity; FVC%: percentage of predicted FVC; FEV_1_: forced expiratory volume in the first second; FEV1%: percentage of predicted FEV_1_; FEV_1_/FVC: ratio FEV1/FVC; P_Imax_: maximal inspiratory pressure; RPE: Borg’s perceived exertion; ONB: test free oronasal breathing; FB: test restricted nasal breathing with FeelBreathe.

**Table 2:** Descriptive values and number of responders and non-responders by cardioventilatory variables.

Variables	FB			ONB			Responders	
Tin (s)	1.4	±	0.6	1.0	±	0.3	13	(72.2%) *
RER	0.8	±	0.1	0.9	±	0.1	12	(66.7%)
Tin/Tot (%)	44.7	±	7.2	41.3	±	5.0	10	(55.6%)
Tex (s)	1.7	±	0.6	1.5	±	0.3	9	(50.0%)
B_F_ (breaths/min)	21.2	±	6.7	25.1	±	5.2	9	(50.0%)
EqO_2_ (L/min)	26.5	±	4.2	28.4	±	4.0	9	(50.0%)
F_E_O_2_ (%)	16.6	±	0.7	17.0	±	0.5	9	(50.0%)
Tex/Tot (%)	55.3	±	0.07	58.7	±	0.06	9	(50.0%)
VTex (L)	1.6	±	0.5	1.4	±	0.4	8	(44.4%)
VTin (L)	1.5	±	0.5	1.4	±	0.4	8	(44.4%)
F_E_CO_2_ (%)	3.2	±	0.6	3.0	±	0.5	7	(38.9%)
P_ET_CO_2_ (mmHg)	4.7	±	0.5	4.6	±	0.5	7	(38.9%)
VO_2_ (mL/min)	1130	±	300	1110	±	360	7	(38.9%)
P_ET_O_2_ (mmHg)	14.4	±	0.8	14.6	±	0.6	7	(38.9%)
V_E_ (L/min)	32.1	±	9.0	34.5	±	10.2	7	(38.9%)
EqCO_2_ (L/min)	31.7	±	4.5	33.4	±	4.5	6	(33.3%)
HR (beats/min)	109.2	±	17.0	108.9	±	15.4	5	(27.8%)
VD/VT (L/min)	24.5	±	8.8	26.9	±	8.4	5	(27.8%)
VCO_2_ (mL/min)	900.2	±	343	895.6	±	364	2	(11.1%)

Descriptive values are shown as mean (standard deviation). Responders/non-responders are shown as number (percentage). * *p* < 0.05 for one-sample proportion test. Tin: inspiration time; Tin/Tot: inspiratory time fraction; VTex: expiratory tidal volume; F_E_CO_2:_ fraction of expired carbon dioxide; VO_2_: oxygen uptake; Tex: expiration time; VTin: inspiratory tidal volume;VCO_2_: carbon dioxide production; HR: heart rate; P_ET_CO_2:_ end-tidal partial pressure of carbon dioxide; F_E_O_2_: fraction of expired oxygen; Tex/Tot: expiratory time fraction; B_F_: breathing frequency; V_D_/V_T_: deadspace/tidal volume ratio; RER: respiratory quotient; EqCO_2:_ ventilatory equivalent ratio for carbon dioxide; EqO_2_: ventilatory equivalent for oxygen;PETO_2_: end-tidal partial pressure of oxygen; V_E_: minute ventilation.

## Supplementary Materials

The following are available online at https://www.mdpi.com/1660-4601/17/13/4876/s1, Figure S1: Feel Breathe Mod. COPD-4 mm. Patent Number: P200902402, Figure S2: Feel Breathe in COPD patient. FB is placed partially covering the nostrils. It is made with hypoallergenic tissue and sweat resistant.

## References

[1] Crisafulli E., Costi S., Fabbri L.M., Clini E.M. (2007). Respiratory muscles training in COPD patients. Int. J. Chron. Obstruct. Pulmon. Dis..

[2] Rycroft C.E., Heyes A., Lanza L., Becker K. (2012). Epidemiology of chronic obstructive pulmonary disease: A literature review. Int. J. Chron. Obstruct. Pulmon. Dis..

[3] Petrovic M., Reiter M., Zipko H., Pohl W., Wanke T. (2012). Effects of inspiratory muscle training on dynamic hyperinflation in patients with COPD. Int. J. Chron. Obstruct. Pulmon. Dis..

[4] Gosselink R., De Vos J., Van Den Heuvel S.P., Segers J., Decramer M., Kwakkel G. (2011). Impact of inspiratory muscle training in patients with COPD: What is the evidence?. Eur. Respir. J..

[5] Geddes E.L., O’Brien K., Reid W.D., Brooks D., Crowe J. (2008). Inspiratory muscle training in adults with chronic obstructive pulmonary disease: An update of a systematic review. Respir. Med..

[6] Bernardi E., Pomidori L., Bassal F., Contoli M., Cogo A. (2010). Respiratory muscle training with normocapnic hyperpnea improves ventilatory pattern and thoracoabdominal coordination, and reduces oxygen desaturation during endurance exercise testing in COPD patients. Int. J. Chron. Obstruct. Pulmon. Dis..

[7] González-Montesinos J.L., Ponce-González J.G., Vicente-Campos D., López-Chicharro J., Fernández-Santos J.R., Vaz-Pardal C., Costa-Sepúlveda J.L., Conde-Caveda J., Castro-Piñero J. (2016). Effects of a nasal ventilator restriction device on lung ventilation and gas exchange during exercise in healthy subjects. Nutr. Hosp..

[8] Morton A.R., King K., Papalia S., Goodman C., Turley K.R., Wilmore J.H. (1995). Comparison of maximal oxygen consumption with oral and nasal breathing. Aust. J. Sci. Med. Sport.

[9] Bailey S.J., Romer L.M., Kelly J., Wilkerson D.P., DiMenna F.J., Jones A.M. (2010). Inspiratory muscle training enhances pulmonary O(2) uptake kinetics and high-intensity exercise tolerance in humans. J. Appl. Physiol..

[10] Faul F., Erdfelder E., Lang A.G., Buchner A. (2007). G*Power 3: A flexible statistical power analysis program for the social, behavioral, and biomedical sciences. Behav. Res. Methods.

[11] Celli B.R., MacNee W., Agusti A., Anzueto A., Berg B., Buist A.S., Calverley P.M.A., Chavannes N., Dillard T., Fahy A. (2004). Standards for the diagnosis and treatment of patients with COPD: A summary of the ATS/ERS position paper. Eur. Respir. Soc..

[12] Uschner D., Schindler D., Hilgers R.D., Heussen N. (2018). randomizeR: An R Package for the Assessment and Implementation of Randomization in Clinical Trials. J. Stat. Softw..

[13] Spruit M.A., Singh S.J., Garvey C., ZuWallack R., Nici L., Rochester C., Hill K., Holland A.E., Lareau S.C., Man W.D.C. (2013). An official American thoracic society/European respiratory society statement: Key concepts and advances in pulmonary rehabilitation. Am. J. Respir. Crit. Care Med..

[14] Hopkins W.G. (2000). Measures of Reliability in Sports Medicine and Science. Sport Med..

[15] Yue Y., Bolin D., Rue H., Wang X.F. (2019). Bayesian Generalized Two-way ANOVA Modeling for Functional Data Using INLA. Stat. Sin..

[16] Lindgren F., Rue H. (2015). Bayesian Spatial Modelling with R-INLA. J. Stat. Softw..

[17] Bolin D., Lindgren F. (2018). Calculating Probabilistic Excursion Sets and Related Quantities Using excursions. J. Stat. Softw..

[18] R Core Team (2007). R: A Language and Environment for Statistical Computing.

[19] Polkey M.I., Moxham J. (2011). Attacking the disease spiral in chronic obstructive pulmonary disease: An update. Clin. Med..

[20] Puente-Maestu L., Abad Y.M., Pedraza F., Sánchez G., Stringer W.W. (2006). A controlled trial of the effects of leg training on breathing pattern and dynamic hyperinflation in severe COPD. Lung.

[21] Hill K., Jenkins S.C., Hillman D.R., Eastwood P.R. (2004). Dyspnoea in COPD: Can inspiratory muscle training help?. Aust. J. Physiother..

[22] Gloeckl R., Marinov B., Pitta F. (2013). Practical recommendations for exercise training in patients with COPD. Eur. Respir. Rev..

[23] Jackson C. (1996). Humidification in the upper respiratory tract: A physiological overview. Intensive Crit. Care Nurs..

[24] Calbet J.A., Losa-Reyna J., Peralta R.T., Rasmussen P., Ponce-Gonzalez J.G., Sheel A.W., de la Calle-Herrero J., Grau A.G., Morales-Alamo D., Fuentes T. (2015). Limitations to oxygen transport and utilisation during sprint exercise in humans: Evidence for a functional reserve in muscle O diffusing capacity. J. Physiol..

[25] Morales-Alamo D., Losa-Reyna J., Torres-Peralta R., Martin-Rincon M., Perez-Valera M., Curtelin D., Ponce-González J.G., Santana A., Calbet J.A. (2015). What limits performance during whole-body incremental exercise to exhaustion in humans?. J. Physiol..

[26] Pertuze J., Watson A., Pride N.B. (1991). Maximum airflow through the nose in humans. J. Appl. Physiol..

[27] Burdon J.G., Killian K.J., Jones N.L. (1983). Pattern of breathing during exercise in patients with interstitial lung disease. Thorax.

[28] Van Meerhaeghe A., Scano G., Sergysels R., Bran M., De Coster A. (1981). Respiratory drive and ventilatory pattern during exercise in interstitial lung disease. Bull. Eur. Physiopathol. Respir..

[29] Harty H.R., Corfield D.R., Schwartzstein R.M., Adams L. (1999). External thoracic restriction, respiratory sensation, and ventilation during exercise in men. J. Appl. Physiol..

[30] Miki K., Tsujino K., Edahiro R., Kitada S., Miki M., Yoshimura K., Kagawa H., Oshitani Y., Ohara Y., Hosono Y. (2018). Exercise tolerance and balance of inspiratory-to-expiratory muscle strength in relation to breathing timing in patients with chronic obstructive pulmonary disease. J. Breath Res..

[31] Fregosi R.F., Lansing R.W. (1995). Neural drive to nasal dilator muscles: Influence of exercise intensity and oronasal flow partitioning. J. Appl. Physiol..

[32] Stanescu D., Pattijn J., Clement J., Van de Woestijine K.P. (1972). Glottis opening and airway resistance. J. Appl. Physiol..

[33] Somfay A., Pórszász J., Lee S.M., Casaburi R. (2002). Effect of hyperoxia on gas exchange and lactate kinetics following exercise onset in nonhypoxemic COPD patients. Chest.

[34] Katayama K., Iwamoto E., Ishida K., Koike T., Saito M. (2012). Inspiratory muscle fatigue increases sympathetic vasomotor outflow and blood pressure during submaximal exercise. AJP Regul. Integr. Comp. Physiol..

[35] Boeselt T., Nell C., Lütteken L., Kehr K., Koepke J., Apelt S., Veith M., Beutel B., Spielmanns M., Greulich T. (2017). Benefits of high-intensity exercise training to patients with chronic obstructive pulmonary disease: A controlled study. Respiration.

[36] Langer D., Charususin N., Jácome C., Hoffman M., McConnel A., Decramer M., Gosselink R. (2015). Efficacy of a novel method for inspiratory muscle training in people with chronic obstructive pulmonary disease. Phys. Ther..

[37] McNamara R.J., McKeough Z.J., Mo L.R., Dallimore J.T., Dennis S.M. (2016). Community-based exercise training for people with chronic respiratory and chronic cardiac disease: A mixed-methods evaluation. Int. J. Chron. Obtruct. Pulmon. Dis..

[38] O’Connor C., Lawson R., Waterhouse J., Mills G.H. (2019). Is inspiratory muscle training (IMT) an acceptable treatment option for people with chronic obstructive pulmonary disease (COPD) who have declined pulmonary rehabilitation (PR) and can IMT enhance PR uptake? A single-group prepost feasibility study in a home-based setting. BMJ Open.

[39] Arnedillo A., González-Montesinos J.L., Fernández-Santos J.R., Vaz-Pardal C., España-Dominguez C., Ponce-González J.G., Cuenca-García M. (2020). Effects of a rehabilitation programme with a nasal inspiratory restriction device on exercise capacity and quality of life in COPD. Int. J. Environ. Res. Public Health..

