# Supplementary material for: A New Nasal Restriction Device Called FeelBreathe® Improves Breathing Patterns in Chronic Obstructive Pulmonary Disease Patients during Exercise
Source: Int J Environ Res Public Health. 2020 Jul 6;17(13):4876. doi: 10.3390/ijerph17134876 (PMC7370091; doi:10.3390/ijerph17134876)
Supplement: Supplementary file 1 [file ijerph-17-04876-s001.pdf]

A new nasal restriction device called FeelBreathe® improves breathing patterns in chronic obstructive pulmonary disease patients during exercise

Supplemental file 1:

Device Feel Breathe®

This device, of resistive loading, is comprised of a strip of hypoallergenic material (3M S.A. Medical Specialties / O.E.M. Spain) (Figure 1) that is placed and adhered to the nares under the nasal passages (Gonzalez-Montesinos JL, Castro-Piñero J, Mora-Vicente J, Vaz-Pardal MC, Fernandez santos J, Gómez R, 2010) (Figure 2). Modifying the tissue characteristics, size and shape of the dressing allows the creation of several models with different levels of restriction. This impairs the free entry of air through the nose by producing resistance to flow without exerting pressure on the nares or modifying their shape .

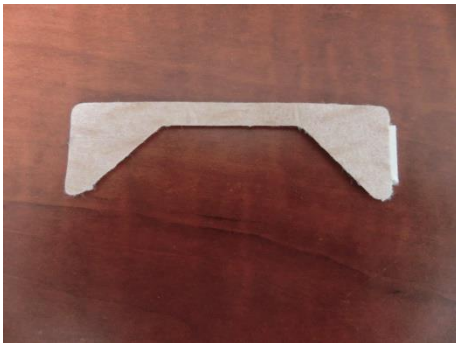

Figure S1: Feel Breathe Mod. COPD-4 mm. Patent Number: P200902402.

Feel Breathe has been authorized by the Spanish Agency for Medicines and Health Products for application on COPD patients (Expedient 521/15/EC. AEMPS-Madrid-Spain).

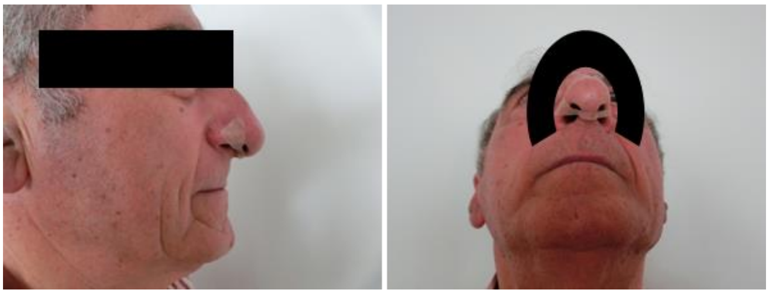

Figure S2: Feel Breathe in COPD patient. FB is placed partially covering the nostrils. It is made with hypoallergenic tissue and sweat resistant.

For restricted nasal breathing FB, the small size was used (FB 4 mm). FB was placed under the nostrils, using sterile gloves and assessing the patient did not have mucus or injuries. During the test execution, patients were allowed to breath only nasal during inspiration, and mouth expiration. If the patient was not able to continue performing only nasal breaths with FB he lifted up his hand and stopped the test immediately. After each exercise, P_{Imax} free oronasal breathing (P_{Imax} Post-ONB), restricted nasal breathing (P_{Imax} Post-FB) and Borg's perceived exertion were examined using a scale from 1-10,and blood pressure was measured before and after performing the tests.

REFERENCES

Gonzalez-Montesinos JL, Castro-Piñero J, Mora-Vicente J, Vaz-Pardal MC, Fernandez Santos J, Gómez R, Costa. J. (2010). P200902402 Nasal Ventilatory flow restriction and filtering device. Universidad de Cádiz-Spain: OEPM.
